# FTT-MA: A Flexible Time-Triggered Middleware Architecture for Time Sensitive, Resource-Aware AmI Systems

**DOI:** 10.3390/s130506229

**Published:** 2013-05-13

**Authors:** Adrián Noguero, Isidro Calvo, Federico Pérez, Luis Almeida

**Affiliations:** 1 Tecnalia Research & Innovation, ICT Division, Parque Tecnológico de Bizkaia, #202, 48170 Zamudio, Spain; 2 Department of Automatic Control and Systems Engineering (DISA), University of the Basque Country (UPV/EHU), Nieves Cano 12, 01006 Vitoria-Gasteiz, Spain; E-Mail: federico.perez@ehu.es; 3 Instituto de Telecomunicações, Fac. de Engenharia, University of Porto, 4200-465 Porto, Portugal; E-Mail: lda@fe.up.pt

**Keywords:** ambient intelligence, ubiquitous computing, middleware architectures, real-time systems, distributed systems, cyber-physical systems (CPS), reconfiguration

## Abstract

There is an increasing number of Ambient Intelligence (AmI) systems that are time-sensitive and resource-aware. From healthcare to building and even home/office automation, it is now common to find systems combining interactive and sensing multimedia traffic with relatively simple sensors and actuators (door locks, presence detectors, RFIDs, HVAC, information panels, *etc*.). Many of these are today known as Cyber-Physical Systems (CPS). Quite frequently, these systems must be capable of (1) prioritizing different traffic flows (process data, alarms, non-critical data, *etc*.), (2) synchronizing actions in several distributed devices and, to certain degree, (3) easing resource management (e.g., detecting faulty nodes, managing battery levels, handling overloads, *etc*.). This work presents FTT-MA, a high-level middleware architecture aimed at easing the design, deployment and operation of such AmI systems. FTT-MA ensures that both functional and non-functional aspects of the applications are met even during reconfiguration stages. The paper also proposes a methodology, together with a design tool, to create this kind of systems. Finally, a sample case study is presented that illustrates the use of the middleware and the methodology proposed in the paper.

## 1. Introduction

Ambient Intelligence (AmI) systems are found in a broad number of domains. Typically, they target smart homes, assisted living, health care systems, shops and business or leisure activities [1]. However, they may also be found in other domains with tighter timing, safety, security or robustness requirements such as transportation systems [2], manufacturing [3] or video security systems [4].

AmI systems involve different operations such as perceiving the state of the environment via several sensors, executing reasoning algorithms to process the sensors data and acting upon the environment using different kinds of controllers. In addition, these operations must be carried out in an adaptive and ubiquitous way [5]. AmI applications rely heavily on communications to coordinate different operations and devices. Even though the use of distribution middleware solutions such as CORBA, ICE, OSGi, OMG DDS or Web Services has been successfully adopted in AmI systems to reduce the distribution complexity [6], most of these middleware technologies are not specifically designed to cope with AmI-specific requirements, such as representing high level abstractions or reconfiguring the applications dynamically. In this scenario the definition of higher level middleware architectures that go beyond the mere resolution of the distribution issues by integrating abstractions to represent system resources, e.g., CPU, network, memory and battery, as well as policies that implement efficient and dynamic resource management algorithms may become a valuable asset for the developers of AmI applications.

This is especially true for AmI applications with timing requirements or with additional resource-related non-functional requirements, e.g., limited resource capacities and resource usage costs, since a broader number of requirements must be met, beyond their functionality, related to latencies, synchronization of distributed operations, Quality-of-Service (QoS) levels, management of physical resources, *etc*. Currently, most of the middleware architectures used in AmI applications do not consider such issues, particularly the combination of support to on-line adaptation and reconfiguration, with timing guarantees that are enforced throughout adaptation/ reconfiguration phases.

Addressing such limitations, this paper presents the Flexible Time-Triggered Middleware Architecture (FTT-MA), going beyond previous works on particular aspects of the architecture [7–10] and providing a broader and integrated view. In addition, this paper presents the methodology associated to using FTT-MA and a sample case study to illustrate how the proposed methodology and middleware architecture can be used to design and develop time-sensitive, resource-aware AmI systems.

FTT-MA is a middleware infrastructure aimed at AmI systems that require: (1) the timely execution of their activities and (2) the flexibility to adapt dynamically according to resource-oriented policies (e.g., load balancing or battery management) or reconfigure at run-time according to changes in the system composition (e.g., joining/leaving nodes or services). It is a time-triggered middleware architecture and, as such, it allows developers to focus on the functionality of the applications separately from synchronization (triggering) and resource management (adaptation) issues. Among time-triggered architectures, this one has the unique feature of supporting reconfiguration / adaptation of the applications at run-time since the scheduling of the time-triggered components is carried out online. Thus, changes in system requirements or composition can be readily accounted for. To the best of our knowledge, this feature is not currently supported by any other time-triggered middleware architecture, which are based on static cyclic schedules.

Moreover, FTT-MA also extends the previous works on FTT protocols since it takes a broader view of the system, integrating a variety of resources that are orchestrated together, as opposed to such protocols that focused only on the network resource, e.g., FTT-CAN [11] or FTT-SE [12].

The flexibility of FTT-MA also extends to the fact that it inherently supports heterogeneous components exchanging heterogeneous data types, such as sensor and control data, video streams and alarm messages, but it also supports the management of replication, scheduling the execution of a set of replicated tasks while optimizing the use of the distributed system resources in order to adapt to changes in the functionality at run-time and cope with transient faults. An implementation prototype of FTT-MA that uses CORBA as distribution middleware is available as open source at [13]; however the same principles could be easily adapted without loss of generality to other distribution technologies, such as ICE or OSGi.

The rest of the paper is structured as follows: Section 2 describes some related work; Section 3 covers the design of FTT-MA; Section 4 describes the methodology used for developing applications with FTT-MA; Section 5 describes an example implementation of the proposed middleware that uses CORBA as distribution middleware, the so-called FTT-CORBA, and it proposes a use case application that eases the understanding and the usage of FTT-MA; finally, Section 6 draws the conclusions and presents some future work.

## 2. Related Work

There are several middleware architectures that address AmI specific requirements. A discussion about the main requirements that middleware technologies must fulfill to adequately support the development of AmI systems can be found in [6]. This work also analyses the use of most popular distribution middleware technologies such as CORBA, ICE and Web Services in this field.

The authors have found in the literature several works that survey existing high-level middleware architectures aimed at providing appropriate software infrastructures for these systems. Most of them, such as [14–17], are built on top of different distribution middleware technologies like CORBA, OSGi, ICE or Web Services. In some cases they address generic domain applications, but in others, they are aimed at specific domains like Ambient Assisted Living [18]. Frequently, they provide abstractions to transparently manage the relevant resources in the physical and virtual spaces, orchestrating the various computational components into a rich, adaptable, flexible and open way. The architectures presented in [16,19] offer high level abstractions for specific domains that hide complexities such as distribution and context awareness. In particular, the work in [19] proposes a formal context model based on four ontologies: users, devices, environment and services. Other works take in consideration the special needs of the underlying network technology; for example, the work in [20] compares the characteristics of several special purpose middleware architectures that execute on top of wireless sensor networks (WSNs).

Other issues must be considered, such as the development and deployment of AmI systems as well as higher-level functionalities. In particular, the work in [21] proposes a software framework that facilitates the development and deployment of AmI scenarios. The work in [22] proposes a service-oriented middleware architecture aimed at allowing service reconfiguration and dynamic integration in ubiquitous systems. This middleware allows building systems that provide the desired functionality by distributing type information of runnable services and interconnecting them as needed.

In the domain of control and real-time systems an expression that is gaining wider acceptance is *Cyber-physical Systems* (CPS). This expression groups several disciplines, mainly real-time systems, network communications and control systems. Basically, CPS integrate embedded computers that control physical processes in different domains, some of which overlap with typical AmI applications such as intelligent buildings, healthcare, transportation systems and factory automation among many others [23,24].

Some authors have provided solutions to cope with the management of timing properties in distributed systems and the use of their infrastructure/resources through classic control theory [25–27] or genetic algorithms [28], focusing on the monitoring and control of certain non-functional properties. Other authors introduced adaptable architectures, typically with flexible mechanisms governed by complex admission control algorithms, to carry out dynamic reconfigurations at run-time [29]. Also, other works focus on the timing synchronization of the applications, by means of time-triggered middleware architectures, but with a limited degree of flexibility [30–32].

Finally, a large number of middleware architectures provide their users, *i.e.*, the designers and developers of the applications, with associated methodologies and tools to foster their adoption (e.g., [33,34]). Many of these tools are based on the Model-Driven Engineering (MDE) paradigm. In those cases where the architectures offer predictable behavior, it is possible to further extend these tools with simulation engines that enable the early validation of the application designs [35,36]. Similarly, for architectures including application management interfaces, it is easy to integrate the management function in user tools specifically designed for application monitoring purposes at run-time [37,38].

In conclusion, to the best of our knowledge, most middleware architectures for AmI systems do not consider timing, including synchronization, and resource-aware issues, implying reconfiguration and adaptation, in an integrated way. Event-triggered architectures address relative timing issues and are inherently flexible. On the other hand, time-triggered architectures focus on time synchronization at the cost of flexibility. In spite of the decades-long discussion thread between these two paradigms, we believe, as in [39], that time synchronization is an enabler mechanism for high performance, confidence and easiness of development in distributed systems. However, flexibility is not less relevant, since its lack may lead to systems that are difficult to repurpose and deploy in new settings, and creating even simple applications typically requires intensive development, adaptation, deployment and management work by experts in a particular infrastructure [40]. Flexibility at run-time is also essential to support resource-management techniques.

This work is inspired by the Flexible Time-Triggered (FTT) paradigm [11,41] and provides a novel time-triggered middleware architecture that offers implicit global synchronization in a flexible setting, providing a holistic solution for the implementation of time-sensitive, resource-aware AmI applications.

## 3. Architecture Description

Previous works on the FTT paradigm initially applied it to control communications, only, directly at the data link layer on top of Ethernet and CAN networks for hard real-time systems. Later, the paradigm was extended to also control tasks in the end nodes, either directly [12,42] or integrated in larger frameworks [43,44]. FTT-MA extends this paradigm applying it on top of different distribution middleware architectures, such as CORBA, ICE, OSGi or Web Services, to orchestrate in time the application activities.

FTT-MA aims at applications that are mostly periodic, even though aperiodic operations can be included, too. More specifically, FTT-MA: (1) ensures the time synchronization of the operations in distributed applications, (2) allows the coexistence of different distributed applications over the same infrastructure (devices and network), (3) manages the communications by using several priority levels and (4) monitors and manages the physical resources (e.g., CPU, memory, battery and network) available in a distributed system. Finally, FTT-MA also (5) provides flexibility to carry out changes of the applications at run-time and (6) eases the implementation of fault-tolerant applications by managing replicated services and devices.

According to the FTT paradigm, time in FTT-MA is an infinite sequence of fixed duration time slots called Elementary Cycles (EC). The EC parameter defines the time granularity in the distributed system. Therefore, the EC must be selected carefully at design time taking into account the timing requirements of the applications to be executed since it cannot be adjusted on-line. A short EC will provide a fine temporal granularity and facilitate the adjustment of the timing parameters during reconfiguration/adaptation phases. On the other hand, a longer EC will incur in a lower associated overhead. Note that there is signalling traffic sent every EC and the percentage of the EC effectively available for data exchange is smaller for shorter EC durations. These overheads are platform-dependent and practical prototypes have been built with EC durations ranging from few to tens of milliseconds.

**Figure 1.**
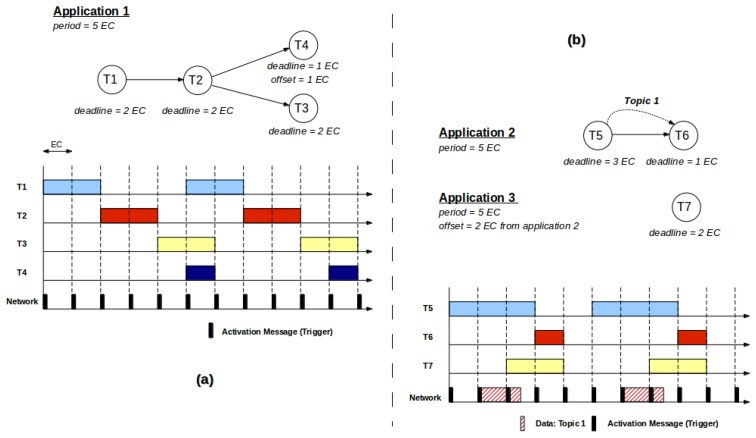
(**a**) Example application graph and time execution diagram including several sequential tasks; (**b**) Example graphs and execution diagram of applications including data exchange and inter-application decoupling.

Applications in FTT-MA are composed of a sequence of ordered tasks, whose order is defined by a directed graph (see Figure 1). Each task (*i.e.*, each node in the graph) is defined in FTT-MA as the minimum executable entity used to compose applications. Tasks are generally considered ubiquitous and can be deployed in any node of the system and, furthermore, the same task could be replicated in several nodes in order to improve fault tolerance.

**Figure 2.**
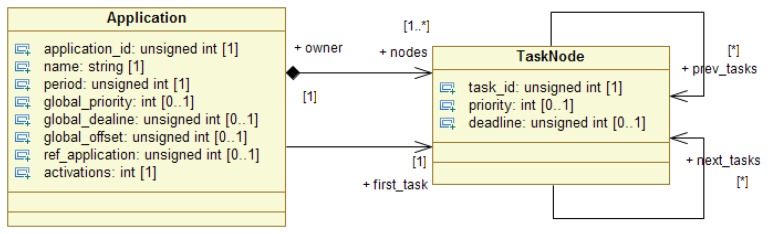
FTT-MA application graphs model in UML

Each task is parameterized with its timing requirements, e.g., deadline and offset. FTT-MA uses this information to generate the task activation triggers as shown in Figure 1. Tasks, *i.e.*, the functional entities of the applications, must not be confused with task instances (or just instances for simplicity), which are the implementations of their functionality deployed in a particular device. Timing parameters, depicted in Figure 2, such as period and offset must be defined at application level. Offsets are used to decouple applications, e.g., two applications with the same period that should not execute simultaneously, and they are very important when a reconfiguration occurs, as it enables the architecture to correctly order the activation of each task. Offsets must always be referred to another application. Aperiodic tasks, common in AmI systems, are introduced using the “*activations*” parameter, which defines how many times an application must execute. This information is provided by an integer number which defines the number of activations. A negative value is understood as a fully periodic application. It is important to remark that every parameter related to time in FTT-MA is always defined in terms of the EC, as shown in the figure.

FTT-MA is designed to operate on top of LANs capable of doing physical multicast, such as Ethernet or IEEE802.11. Actually, it uses multicast messages to activate the tasks instances in the devices of the distributed system, emulating a software bus. Furthermore, FTT-MA provides a particular service, the so-called FTT-Event Channel, to decouple task activations and data messages produced by the tasks. This service avoids collisions in the software bus at run-time and ensures synchronism when a shared communication medium is used to connect the distributed system nodes such as IEEE802.11.

**Figure 3.**
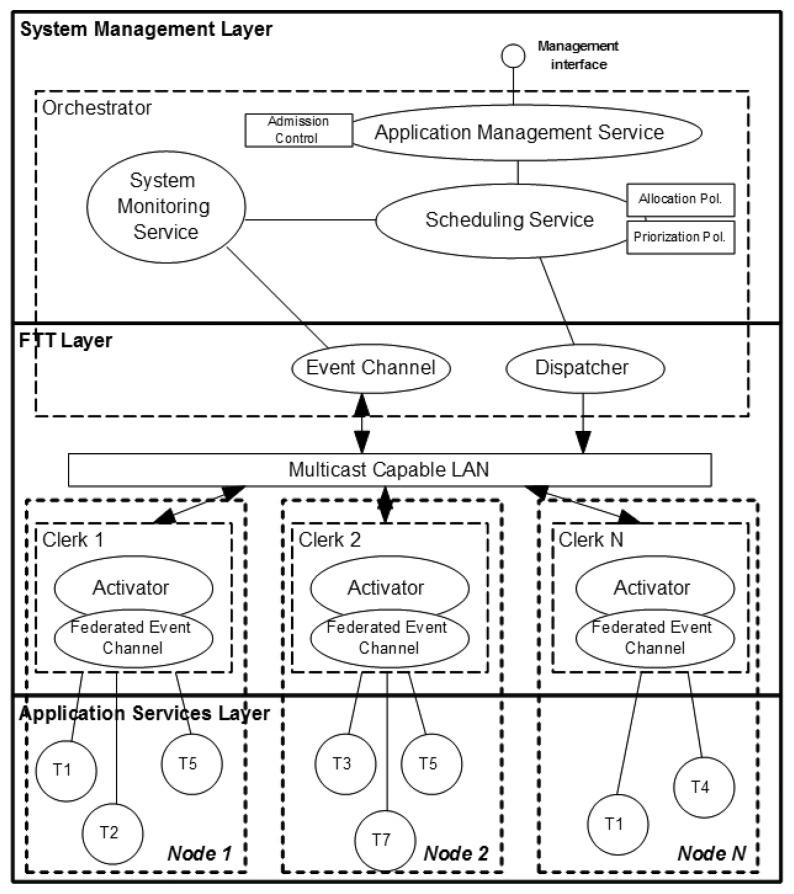
FTT-MA architecture.

The architecture has been designed as a set of services that collaborate among them (see Figure 3). Some services are centralized, *i.e.*, there exists a single instance in the architecture at run-time, whereas other services are distributed over every participant node, which are mostly embedded computers. As depicted in the figure, all centralized services are typically implemented as a single executable: the so-called Orchestrator. Similarly, all services executed at the distributed devices are locally grouped in the so-called Clerk which is another executable.

The proposed architecture is composed of three layers: (1) the System Management Layer which elaborates on-line the execution plan that the FTT Dispatcher enforces, (2) the FTT Layer which triggers the operations of the distributed system and manages the access to the data distribution channel, and (3) the Application Services Layer which implements the services that provide the functionality of the system, e.g., as CORBA methods.

### 3.1. System Management Layer

This layer executes the centralized services devoted to the management of the distributed system and the interaction with the users. More specifically, the services included in this layer are: (1) the Application Management Service (AppMan), (2) the System Monitoring Service (SysMon) and (3) the Scheduling Service (SchedSer).

AppMan provides an interface to allow loading and unloading applications at runtime, modifying the parameters of a running application and monitoring the status of both applications and distributed nodes.

Whenever the AppMan receives a request, the model of the loaded applications is updated, and the new specifications are delivered to SchedSer. AppMan can also execute an admission control test to analyze how the change would affect the behavior of the applications and authorize or reject it in order to avoid unstable or overload situations. The use of the admission control module is optional and the algorithms it executes depend on the working policies selected for a specific system. Section 3.5 discusses briefly some simple admission control policies that have been implemented in the current prototype.

SysMon is responsible for gathering information related to the status of the distributed system nodes. More specifically, this service collects two kinds of data: (1) related to the deployment of the system, *i.e.*, in which distributed nodes the task instances are deployed, and (2) related to the physical status of the node, in terms of CPU consumption, available memory and battery level. This information is provided to the scheduling service SchedSer for the generation of the tasks activation table that the Dispatcher will enforce. SysMon is also capable of detecting inoperative nodes in the distributed system, generating alarms that trigger a rescheduling of the tasks activation table.

Finally, as referred above, SchedSer is responsible for generating the tasks activation table that executes the distributed applications. The SchedSer is triggered when there is a modification in the applications or the SysMon detects any event, e.g., a device has a low battery level. In both cases, the scheduling process is performed in two phases: (1) allocation and (2) prioritization. As outcome of the rescheduling process, SchedSer generates a new table defining in which moment each task instance has to be activated and its priority level. This table will be used by the Dispatcher at the underlying FTT layer to synchronize the activation of the distributed task instances. Tasks period adjustment is used to enforce an upper bound on the activations table.

Since some tasks may be replicated in more than one device during the allocation phase, SchedSer selects which task instances to activate from all instances available that provide the same functionality in a specific system. This process is performed automatically according to an allocation policy selected by the user, e.g., using the node with the highest battery level, keeping a balanced battery level at all nodes or selecting the available node with the highest processing capacity. In FTT-MA, the allocation policy has been designed as a pluggable component, allowing users to select from several alternatives according to the requirements of their system and applications. This is also an extensible approach that eases the implementation of new allocation policies that optimize the usage of the system resources in specific situations.

After allocation, the prioritization phase assigns to every task a priority level at the executing node. Similarly to the allocation policy, the prioritization policy is also a pluggable element, *i.e.*, the user can select a policy from a list or even develop new prioritization policy algorithms and plug them into FTT-MA.

### 3.2. FTT Layer

The FTT layer is responsible for synchronizing the distributed tasks instances and the applications, managing not only the activation of the tasks instances themselves, but also the data exchange among them. This layer is located between the centralized management services and the services located at the distributed nodes. The FTT layer is comprised of four services, two centralized services located at the Orchestrator: the Event Channel and the Dispatcher, and two services replicated in each distributed node: the Federated Event Channel and the Activator. The services in this layer use the multicast software bus.

The Dispatcher, along with the Activators, is responsible for triggering the tasks of the distributed system in a synchronous way enforcing the task activation table provided by SchedSer. This table indicates the Dispatcher (1) the time lapse until the next activation of each task instance, (2) the priority associated to that instance in the distributed node, (3) the execution period and (4) the remaining number of activations, if applicable. This table is used by the Dispatcher until SchedSer generates a new table upon modifications in the functionality or an event is detected by SysMon.

The Dispatcher executes periodically every EC. During each EC the Dispatcher reduces the timer (in EC units) for the next activation of each task instance in one unit. If this timer reaches 0, the dispatcher selects that instance for activation in the current EC. Should the task need to be executed again, the Dispatcher will update the timer for the next activation using the period (in EC units) associated to the application that task is part of.

After selecting the task instances that must be activated, the Dispatcher sends a multicast message to all the Activators in the distributed system with their identifiers and priority values. Activators, which are executed at the distributed nodes, receive these messages and check whether the identifiers in the activation message match any of the task instances deployed in their respective node. In such case, the activator executes the task instance at the designated priority level.

Most frequently, distributed tasks need to share data among them in order to fulfill their functionality, for example, a sensor that acquires data sends this information to a controller. In CPU-bound applications, *i.e.*, those in which the time spent communicating is much less than computing, it is generally acceptable to disregard communications. This is a valid assumption in many applications that use high speed communication links, like in switched Ethernet, to transfer small amounts of data generated at relatively low rates. However, when the communication time is relevant (e.g., in multimedia systems) or when a shared network is used (e.g., IEEE802.11), data messages may collide with activation messages, degrading the synchrony of the whole system. The Event Channel along with the Federated Event Channels of the FTT layer provide a solution to this problem by decoupling activation and data messages through time separation, as depicted by Topic 1 exchanged among T5 and T6 in Figure 1(b).

Messages in the Event Channel are sent using a bottom-up approach. In other words, when a task instance has data to share, it sends a data token to the Federated Event Channel, which is executed locally. To identify different kinds of data tokens, each data token must refer to a topic identifier. The Federated Event Channel holds all data messages that need to be sent. Similarly, if a task instance wants to receive a message, it subscribes to a topic ID in the Federated Event Channel, specifying the kind of data tokens it is expecting to receive.

Since the Event Channel and the Dispatcher are centralized in the Orchestrator, they share the same clock. Thus, the Event Channel may place data messages in the interval between two consecutive activation messages avoiding collisions that may affect synchronization. In order to schedule the transmissions, the Event Channel first polls the Federated Event Channels at the distributed nodes for data tokens in their output queues. After polling, the Event Channel orders the messages according to the priority of the different topics. Finally, the Event Channel commands the Federated Event Channels to send their data tokens, using reliable multicast messages [45]. The number of EC between two consecutive polling processes is called the polling period, which is a configurable parameter in FTT-MA. It is important to note that the FTT-Event Service allows dealing with different levels of criticality, since priorities may be assigned to the topics and it is the Orchestrator who decides according to its priority the order in which topics will be sent.

**Figure 4.**
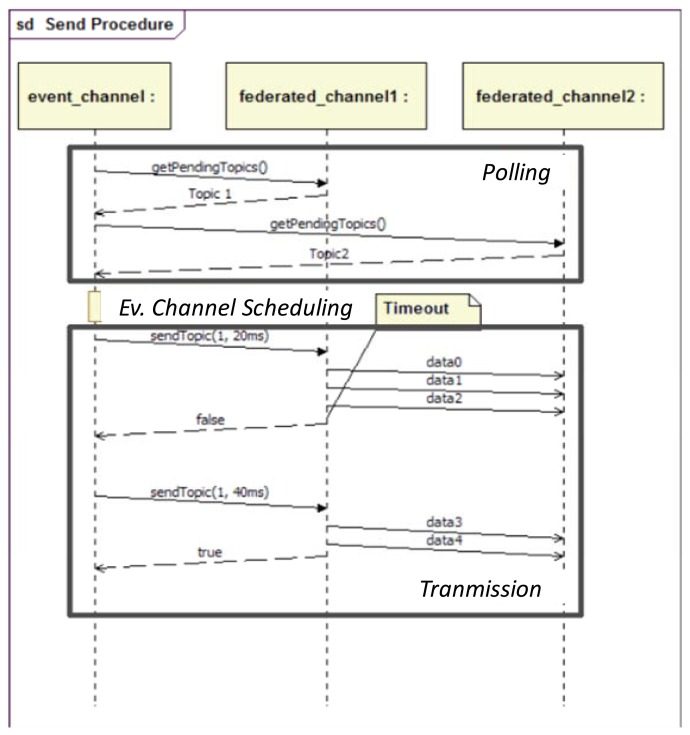
Event channel protocol.

Also, the Event Channel calculates the remaining time until the next activation message and sends it to the Federated Event Channel along with the command to start sending a message. If the Federated Event Channel is able to send the full message before time expires, it notifies the Event Channel, who will select which is the next message to send. Otherwise, the Federated Event Channel notifies of the failure to the Event Channel, and the unsent part of the message (unsent packets) is kept in standby until the next EC (see Figure 4).

The FTT layer is the most important layer of the architecture, providing determinism, as it enables not only the timely and synchronous execution of distributed applications, but also the synchronous distribution of data tokens among tasks, following a fixed priority policy.

### 3.3. Application Services Layer

The instances of the tasks that compose the distributed applications are located at the Application Services Layer. Each task instance is an implementation of a task deployed at a particular distributed node. The technology used to implement task instances must provide mechanisms to ensure that the Activators can execute them and modify their priority according to the requirements.

### 3.4. Architecture Intrinsic Applications

FTT-MA allows the execution of special applications that provide specific services to the architecture, the so-called Architecture Intrinsic Applications. These applications, similar to the services and daemons executed in operating systems, share the execution environment at the distributed nodes with the applications loaded by the users. Consequently their execution must also be scheduled. However, these applications execute specific task instances implemented directly at the Activators and are activated by means of reserved task identifiers.

The current version of FTT-MA defines two Architecture Intrinsic Applications: monitoring and time synchronization. The monitoring application orders the Activator to gather the current status of the physical resources at the distributed nodes, *i.e.*, CPU consumption, available memory and battery level, in order to send it to SysMon. This information is sent using the federated event channel.

The time synchronization application is used to synchronize the clocks of all the distributed nodes. This application sets the time of the clocks of all the distributed nodes to the time of the central clock executed at the Orchestrator.

### 3.5. Admission Control

It has already been mentioned that FTT-MA offers an optional admission control mechanism to prevent that changes in the requirements lead to an invalid execution of the system, e.g., violating QoS parameters of the applications such as bandwidth or deadlines. This module is part of the application management service that interacts with the users and can be turned on or off as desired.

The admission control module takes advantage of the centralization of SysMon to access the status information of the distributed nodes. It uses this information to analyze the stability of the system with the new configuration in terms of schedulability, and availability of memory and bandwidth. The admission algorithms are based on a mathematical model, such as that described in detail in [7]. Since SysMon is capable of tracking down changes in CPU usage and memory, the algorithm computes the current computational load and memory used at every node and anticipates whether introducing a new application will surpass the thresholds defined for every node. Regarding network resources, the admission control algorithm proposed in [7] applies to distributed systems that use the Event Channel over a shared communication network, only.

### 3.6. Implementation Requirements

The implementation of FTT-MA imposes three main requirements on the underlying technology: (1) task encapsulation, (2) availability of fixed priority scheduling mechanisms at the distributed nodes and (3) availability of reliable multicast communications.

Task encapsulation is required to treat task instances as individually executable entities. Encapsulation is a common characteristic in object-oriented languages, such as Java, C++ or C#, as well as in object-oriented, component-oriented, or service-oriented middleware architectures, such as CORBA, ICE or Web Services.

Fixed priority schedulers are needed at the distributed nodes whenever the FTT-MA prioritization module is used. This module may use several policies to assign each task instance in an application a specific priority level according to its timing requirements. These priority levels are sent to the Activators, who should execute the task instance at the priority level provided by SchedSer. This is not a restrictive requirement, since fixed priority schedulers are available in most operating systems as well as in many virtual machine engines, such as Java.

Finally, communications inside FTT-MA have been designed following the software bus paradigm, where remote nodes are capable of receiving the commands from the network master with small jitter values. This kind of communications requires the support for physical multicasting in the network. Moreover, it is important to prevent messages from being lost; thus, the reliable multicast protocol [45] has been selected as communication protocol inside FTT-MA.

## 4. Development and Deployment of FTT-MA Applications

This section briefly presents a methodology aimed at fully exploiting the capabilities of the presented architecture. This methodology, which eases the development and deployment of FTT-MA systems, is supported by a tool developed by the authors, the so-called FTT-Modeler [8].

**Figure 5.**
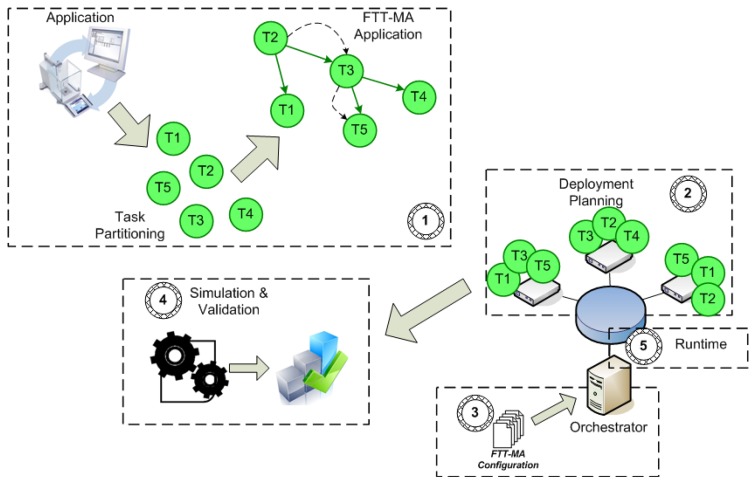
Methodology for application development and deployment.

As depicted in Figure 5, the proposed methodology is split into five phases: (1) application partitioning into tasks, (2) task instances deployment, (3) configuration of FTT-MA parameters, (4) simulation and early validation of the design, and (5) implementation and runtime monitoring. FTT-Modeler supports the five phases of the proposed methodology and automates some stages by means of Model Driven Engineering (MDE) techniques.

The first phase of the methodology consists in partitioning the functionality of each application into individually executable entities exploring inherent concurrency. The criterion to guide the partitioning process may vary depending on the applications; nevertheless, it is possible to identify a set of golden rules that may guide the designer. For example, if a part of an application is physically linked to the hardware, e.g., acquiring data from a sensor, that part should become a task. Similarly, if a specific functionality can be parallelized to improve performance that functionality should also become a task. The same is also valid for critical functionality that can be replicated in the system to improve fault tolerance. Finally, a functionality that is used several times by one or many applications should also become an individual task, as this will improve the maintainability of the system.

**Figure 6.**
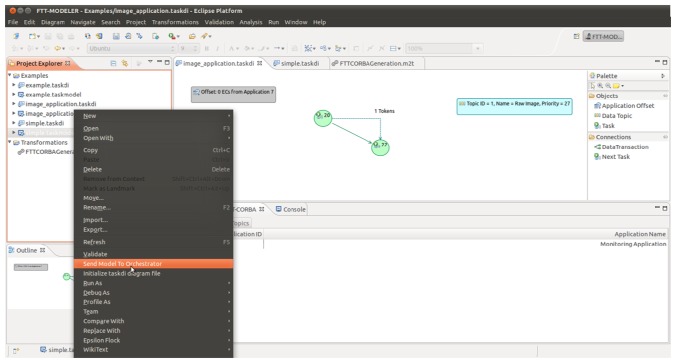
Screenshot of the FTT-Modeler tool.

Once tasks have been extracted, applications are weaved together by establishing the precedence relationships between tasks, messages and their timing properties, *i.e.*, application periods, offsets, deadlines, *etc*. The result of this phase is a set of acyclic directed graphs that define the distributed applications of the distributed system, similar to those shown in Figure 1. The FTT-Modeler tool provides a specific editor for the creation of these graphs developed using MDE techniques. A screenshot of the tool is shown in Figure 6.

The second phase of the methodology is devoted to the deployment of the distributed system. In FTT-MA, this refers to the allocation of the task instances to the nodes of the distributed system. In this process it is important to consider the dependencies that some tasks have with respect to specific nodes, e.g., a sensing task must be deployed in a node with a particular sensor or hardware. Furthermore, tasks intended to be executed in parallel or as back-up instances should be deployed in different nodes.

The third phase aims at selecting the configuration parameters of the middleware architecture, namely, the EC, the polling period and the allocation and prioritization policies to use. The selection of these parameters varies according to the applications; for example, an EC value of 40 ms could be well suited to support a video application that shows a video stream to the users with a rate of 25 frames per second. However, if a 5 ms feedback control application is to be supported together with the referred video stream then an EC of 5 ms should be used. In practice, the EC should be set to the largest value that allows expressing periods and offsets of interest to the system as integer multiples of such value. The FTT-Modeler tool also provides an editor for the creation of deployment models, including the parameterization of the FTT-MA architecture.

FTT-MA has been designed to be deterministic as typical time-triggered architectures. As a consequence, it is possible to simulate its behavior once the application and deployment models are ready. This is the focus of the fourth phase being devoted to the simulation and early validation of the designed applications. The simulation engine implemented in FTT-Modeler allows designers to obtain information from the designs and foresee how the distributed system will behave at runtime with good accuracy.

Once the initial design has been validated, task instances must be implemented and deployed on the nodes according to the plan. Typically, the implementation of a task instance will involve some structural code that can be easily converted to a template and automatically generated, e.g., creating a new CORBA object or a new Java class extending Thread. Since the FTT-Modeler tool has been implemented using MDE techniques, it enables the generation of the structural code of a task instance as well as start-up scripts using model-to-text transformations. Currently, FTT-Modeler is capable of generating the skeleton for task instances compatible with a specific implementation of FTT-MA that uses CORBA as distribution middleware, called FTT-CORBA (see Section 6). The behavior of the distributed system can be monitored using the interface provided by AppMan, in the system management layer.

## 5. FTT-MA Implementation and Use Case

### 5.1. FTT-CORBA

Currently, there is an FTT-MA implementation available, known as FTT-CORBA [13], capable of activating tasks wrapped as CORBA objects, but the same principles could be adapted to be used within other distribution middleware specifications, such as ICE, OSGi or even OPC, without loss of generality. CORBA has been selected for being an open standard and for presenting some support to real-time applications. Furthermore, despite its maturity, CORBA is still considered a valid technology in the AmI domain [2,6,16].

In order to validate the proposed middleware architecture, the authors implemented FTT-MA using CORBA/CORBA-RT as underlying technology, the so-called FTT-CORBA [9]. It has been built on top of ACE and TAO [46]. TAO is an open source CORBA ORB that has a very good performance on devices with limited resources, and the ACE library provides a good abstraction layer that fosters the portability of the implementation to many operating systems. The selected technology fulfills the three requirements imposed on the technology in Section 3.6. Namely: (1) CORBA enables the encapsulation of tasks as CORBA methods, (2) using the CORBA real-time extensions the execution priority of a task can be selected remotely, and (3) the ACE library implements the UDP reliable multicast protocol.

The FTT-CORBA implementation is comprised of two executables: the Orchestrator and the Clerk, which implement the centralized and decentralized services of the FTT-MA architecture respectively. In addition to the decentralized services, the Clerk also implements the connection with the task instances.

The external interfaces of both Orchestrator and Clerk have been implemented as standard CORBA methods (e.g., the Application Management Service interface), while interfaces between internal services have been implemented using shared data structures. CORBA requires that a client that invokes a method knows in advance the stubs of the method, for that reason during start-up Clerks have to create the links to the task instances deployed at their distributed node by using the CORBA DII (Dynamic Invocation Interface), which requires the name of the CORBA object containing the task instance, the name of the CORBA method where the task instance is implemented and any parameters that should be provided to the method. All this information is loaded into the Clerks using configuration files generated by the FTT-Modeler tool.

It is important to note that FTT-MA allows combining several technologies to activate the tasks of the distributed system by means of Clerks that use different technologies (e.g., a Clerk could activate CORBA objects whereas another could activate an OPC component or even an ICE object). Furthermore, the selected implementation technology impacts the performance of the architecture itself. As for FTT-CORBA, the authors have measured the performance of the middleware in terms of task activation jitter for periodic tasks and communication latencies. The results of the laboratory tests [10] demonstrated that FTT-CORBA introduced small jitter in the system (∼600 μs) and that it succeeded in reducing the average latencies of high priority communication messages.

### 5.2. Case Study: A Train Monitoring System

This subsection presents a simple synthetic case study aimed at showing the capabilities of FTT-MA. It does not only illustrate how the FTT-MA middleware works, but also the methodology users should follow in order to develop applications on top of FTT-MA.

The proposed case study uses FTT-MA as backbone for the implementation of an information monitoring system in trains. The TCN standard [47] divides hierarchically train communications at two different levels: vehicle and train. At the vehicle level, the Multifunction Vehicle Bus (MVB) provides the communication infrastructure for low level hard real-time control processes that take place inside the vehicles. Most of these processes are related to the dynamics of the vehicle, such as braking or suspension, and are time-critical. Also, TCN defines a higher level, the train bus level known as Wire Train Bus (WTB), which interconnects all vehicles and enables the interaction for less time critical control processes (e.g., related to ambient information such as door sensors, smoke sensors, video applications, *etc*.). With regard to the communication technologies, although field buses are preferred at vehicle level, switched Ethernet is becoming increasingly popular for the implementation of train level TCN communications [48].

This case study illustrates how FTT-MA may be used as middleware for building train wide applications. It focuses on the six objectives previously mentioned in this article, namely: (1) time synchronization of the operations in a distributed system, (2) graceful coexistence of several distributed applications over the same infrastructure (network and devices), (3) coexistence of different kinds of communication traffic (*i.e.*, periodic, aperiodic, synchronous and asynchronous), (4) physical resource monitoring and detection of unavailable nodes, (5) reconfiguration of the applications at run-time, and (6) implementation of fault-tolerance, parallel computation and resource management policies (e.g., load balancing). The proposed case study is also intended to demonstrate the FTT-MA methodology, briefly described in Section 4, by means of a hypothetical application. Thus, each of the phases defined in the FTT-MA design and development methodology will be explained in detail.

**Figure 7.**
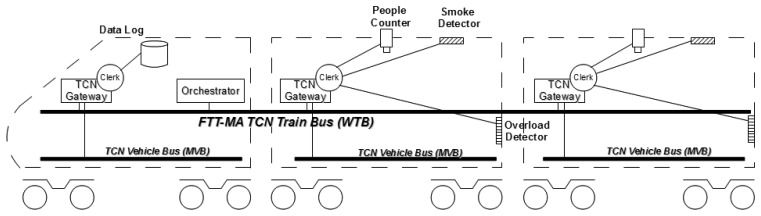
FTT-MA governed TCN bus.

The proposed case study system is aimed at logging train status information in a database. As shown in Figure 7, every train vehicle includes a local TCN bus (MVB) for hard real-time control operations, mainly related to the vehicle dynamics, and a TCN gateway node connected to the WTB. In addition to this, each passenger vehicle has been equipped with a video server capable of counting the people inside each vehicle, as well as sensors to detect ambient information in the vehicle, e.g., smoke and overload detectors. In this context, the data logging application stores periodically the status of the TCN vehicle bus sensors, as well as the number of passengers and the status of the smoke and overload detectors when alarms occur inside the vehicles. In order to get additional information on the behavior of the passengers, the data logging application is able to store video streams coming from the video server whenever an alarm of any kind is detected. However, video is not continuously recorded in order to reduce the data storage requirements but, instead, the system is required to reconfigure itself when any alarm occurs.

Figure 7 also shows the integration of the FTT-MA entities in the system, namely, a dedicated node holding the Orchestrator which is connected to the train bus for the management of the task synchronization and the communications of the system; and a Clerk which is deployed along with each TCN gateway node at every train vehicle. This TCN gateway has been connected to the soft real-time elements of the vehicle; this is, the video server including the people count algorithm and the smoke and overload detectors. TCN gateways are connected together via a dedicated Switched Ethernet over which FTT-MA emulates a soft real-time bus.

#### 5.2.1. Phase 1: Application Partitioning

The proposed case study has been split into several FTT-MA applications and tasks. All FTT-MA applications and tasks that comprise the case study; along with their characteristics and time parameters are described in Tables 1 and 2.

**Figure 8.**
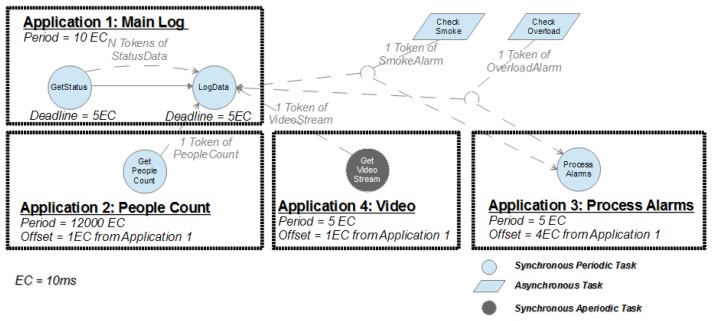
Graphs of the TCN applications.

As shown in Table 1 and Figure 8, the use case has been partitioned into four applications, namely: (1) Main Log, (2) People Count, (3) Process Alarms and (4) Video. These applications involve three different types of tasks: (1) synchronous periodic tasks (*GetStatus*, *LogData*, *GetPeopleCount* and *ProcessAlarms*), which are continuously activated by FTT-MA, (2) synchronous aperiodic tasks (*CheckSmoke* and *CheckOverload*), which are activated by FTT-MA, but may not be active continuously, and (3) asynchronous tasks (*GetVideoStream*), which are not activated by FTT-MA, but produce data that is managed by the FTT Event Channel.

Application tasks have been organized into four, simple, FTT-MA applications, as depicted in Figure 8. In this case, the application graph includes not only the representation of the application, but also the data exchanged among the tasks. This graph also shows asynchronous tasks for the sake of completeness, in order to facilitate the understanding of the case study. However, these tasks are not triggered by FTT-MA, since their activation is not handled by the middleware but they are activated by the occurrence of alarms.

One of the most relevant characteristics of FTT-MA is the capability of synchronizing the activation of several tasks at several distributed devices according to a predefined schedule prepared by SchedSer. However, when several tasks are triggered simultaneously, the responsibility of handling their effective activation is delegated to the operating system at the distributed devices. Nevertheless, FTT-MA allows using the application reference offsets as design parameters to de-phase the applications activations, adding more real-time control to the distributed system. The correct selection of the applications reference offset values, which impose concrete offsets to their tasks according to their specified relative offsets, is an important issue in the configuration of FTT-MA systems. Whenever application offsets are required, an FTT-MA application must also be selected as time reference for the offset definition of the remaining applications. In this case study application, the *Main Log* application was selected as offset reference. Other applications such as *Get People Count* and *Process Alarms* need to be de-phased from *Main Log* using offset values of 10 ms and 40 ms, respectively. This way the pace of execution triggers will never lead to a concurrent execution of any of the tasks in the distributed nodes. Regarding the Video application, the selection of the offset value and reference application is even more important, since this application is dynamically loaded/removed from the distributed system depending on the alarms detected by the sensors. In this case study, the offset value serves not only for de-phasing applications, but also for establishing the moment when the Video application should start executing whenever it is loaded.

#### 5.2.2. Phase 2: Deployment Planning

Once distributed applications, along with their composing tasks, have been defined, the next step is planning the number of instances of each task to be deployed at every device of the distributed system. Different strategies for the deployment of the task instances will lead to different middleware behavior; thus, the deployment must be carefully designed. In the proposed case study, tasks are tightly coupled to the nodes due to hardware restrictions; however, not all the tasks are intended to behave in the same way. As shown in Table 2, the *GetStatus* task is deployed to all the vehicles, *ProcessAlarms* and *LogData* are deployed only in the locomotive whereas *GetPeopleCount* and *GetVideoStream* are deployed only in the passenger vehicles.

Some tasks, such as *GetStatus* and *GetPeopleCount*, are intended to be executed simultaneously in all passenger vehicles in parallel. Deployed instances share the same identifier since local Clerks at the distributed devices are activated in parallel by the same trigger message. FTT-MA handles these tasks as a single instance. On the other hand, instances of the *GetVideoStream* task, which are also deployed in the passenger vehicles, need to be different from each other, since they should be activated individually, only if an alarm is detected in the corresponding vehicle. As a result, the Video application, along with the associated tasks, will be formed by different applications in the different vehicles, loaded and removed from the system independently.

Finally, tasks not handled by FTT-MA are deployed in the passenger vehicles independently. It is important to note that, since their activation is not handled by the middleware their execution is expected to be concurrent and asynchronous.

#### 5.2.3. Phase 3: FTT-MA Parameters Configuration

FTT-MA provides several parameters that allow the users to fine tune the behavior of the distributed applications once deployed. These parameters include the EC, the allocation and prioritization policies, and, if applicable, the priorities of the distributed tasks and data topics.

The EC should be set to a common divisor of all the periods of the different tasks extracted from the application requirements. In this case study, considering the time parameters shown in Tables 1 and 2 several EC values could be used, e.g., 50, 25 or 10. An EC value of 10 ms was selected in order to achieve a good balance between time resolution, computing overhead at the distributed nodes and bandwidth overhead at the network.

Allocation and prioritization policies are used to define how the scheduling service of the FTT-MA selects the task instances to execute and how priorities are managed locally at the distributed nodes. This is particularly important when several task instances are deployed in more than one node to implement fault-tolerance or load balancing protocols. The proposed case study does not include any of these requirements; therefore, the default policies are selected, *i.e.*, allocation to the first node available, and fixed priority scheduling in the distributed nodes.

Finally at this phase, priorities must be assigned for each task and each data topic. Task priorities define how the operating systems execute the tasks in case of concurrent execution. Topic priorities define the order in which the FTT Event Channel sends the messages through the network. The priorities selected for the tasks in the case study application are shown in Table 3. Table 4 shows how the priorities of the different data topics have been selected, along with the size of each data token and the estimated transmission time (calculated for a 100 Mbps Ethernet LAN and EC = 10 ms).

#### 5.2.4. Phase 4: Simulation and Validations

Before deploying the distributed applications to the train, an early simulation of the behavior of the system should be done to prevent errors that could be easily corrected in earlier stages. The simulation of distributed applications on top of the FTT-MA middleware can be easily carried out due to the unique characteristics of FTT-MA, particularly, using the EC as main time reference. When the distributed system involves the dynamic reconfiguration of the system, like in this case study, the simulation should also include the events that trigger the reconfiguration so that the final behavior can be studied in detail.

**Figure 9.**
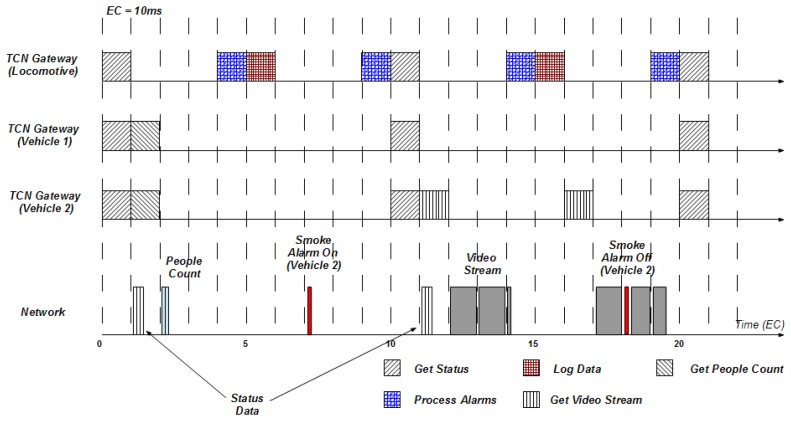
Simulation results for a train including a locomotive and two passenger vehicles.

The results of the use case application simulation, including the locomotive vehicle and two passenger vehicles, are shown in Figure 9. This diagram describes how the system behaves not only during the regular state, but also when an alarm arises and the video streaming application is turned on, according to the configuration parameters set in Tables 1, 2, 3 and 4. As shown in the diagram, the Main Log application, comprised of the *GetStatus* and *LogData* tasks, executes periodically every 10 EC (100 ms). In addition, the diagram shows how the *GetStatus* task executes in parallel in all the vehicles at the same time, because the deployed task instance is the same in all vehicles and, consequently, they are activated by the same trigger message. The diagram also shows the effect of the offset values defined for the application. In particular, an offset of 1 EC (10 ms) and an offset of 4 EC (40 ms) have been assigned to the People Count and Process Alarms applications respectively.

Regarding communications, the diagram depicts the usage of the network, managed by the FTT Event Channel. It is important to note that the FTT Event Channel is capable of preempting low-priority traffic (video stream, in the example) with high priority traffic (alarms). In the diagram the trigger messages sent by the Orchestrator have been omitted for the sake of figure readability; however, activation messages are sent at the beginning of every EC, whenever a task must be triggered. Finally, the diagram provides some information of the dynamic behavior of the system, showing how the Video application (and consequently the *GetVideoStream* task) is dynamically started/stopped when the smoke alarm is turned on/off on vehicle number 2.

#### 5.2.5. Final Considerations

This case study has demonstrated how the FTT-MA architecture can be used for the implementation of synchronous distributed applications. More specifically, the example has shown how FTT-MA is capable of synchronizing applications, enabling its coexistence and preventing collisions. In addition, the example described how different kinds of traffic can be defined and managed by the proposed middleware, and how different execution policies (*i.e.*, parallel or individual) can be established using the deployment strategy and the FTT-MA policies.

The example could be further refined by adding fault-tolerance to the logging application. To do so an extra distributed node could be connected to the WTB and to the database, using a different task instance of the *LogData* task. This way, if a node should become unavailable, FTT-MA would automatically switch to the other node, preventing data from being lost.

## 6. Conclusions and Future Work

In this paper the authors have presented a time-triggered middleware architecture suitable for ubiquitous applications, the so called FTT-MA. This architecture enables developers to focus on the functionality of the applications separately from other issues such as: (1) meeting temporal constraints, *i.e.*, satisfying deadlines, periods, off-sets or synchronizing distributed tasks, (2) using adequately the physical resources, *i.e.*, CPU, memory, battery and network, (3) reconfiguring the functionality of the system at run-time using resource aware policies. FTT-MA can be implemented on top of any distribution middleware platform that fulfills the following requirements: (1) task encapsulation, (2) availability of fixed priority scheduling mechanisms at the distributed nodes and (3) availability of reliable multicast communications. FTT-MA provides the user with a service-based interface to interact with the distributed system at run-time, including mechanisms to load/unload applications, monitor the status of the nodes, and modify the configuration parameters of the system.

The paper also presented a methodology and a tool to help designers and developers of applications to fully exploit the capabilities of the presented architecture. Namely, this tool eases the development and deployment of FTT-MA applications as well as their operation at run-time. Finally, a use case application has been provided to validate FTT-MA and the associated methodology.

Regarding future work, we will address the fault-tolerance of the Orchestrator, which is currently a single point of failure, possibly using a semi-active replication mechanism. Another aspect that we wish to address in future work is the development of gateways to allow the connection of legacy equipment that is not FTT-MA compliant to an FTT-MA system.Currently, there is an implementation of FTT-MA for tasks wrapped as CORBA objects, called FTT-CORBA. This implementation is available as open source software at [13]. We also plan to implement FTT-MA over other middleware technologies namely DDS, ICE and OPC and we plan to apply the proposed middleware to a variety of real application scenarios to further assess the benefits that it brings.

## Figures and Tables

**Table 1.** Description of the FTT applications of the TCN use case.

**Application**	**Description**	**Period**	**Offset**
Main Log	Periodically get the status of all the TCN vehicle buses and store the information in the database. All data gathered by the rest of the applications will also be stored.	100 ms	Ref *
People Count	Get the result of the people count algorithm in a vehicle	2 min	10 ms
Process Alarms	Check if any alarm has been triggered in any vehicle and reconfigures the application	50 ms (when active)	40 ms
Video	Get the next set of video frames recorded by the video server in a vehicle	50 ms	10 ms

* Main Log application is used as reference for offset management.

**Table 2.** Description of the tasks of the TCN application.

**Task**	**Description**	**Location**	**Produced Topics**	**Consumed Topics**
Get Status	Get the last measurements from the sensors connected to the TCN vehicle bus	All vehicles	StatusData	-
Get People Count	Get the result of the people count algorithm in a vehicle	Passenger vehicles	PeopleCount	-
Get Video Stream	Get the next set of video frames recorded by the video server in a vehicle	Passenger vehicles	VideoStream	-
Process Alarms	Check if any alarm has been triggered in any vehicle and reconfigures the application	Locomotive	-	SmokeAlarm OverloadAlarm
Log Data	Store the status of the trains to the database	Locomotive	-	StatusData PeopleCount SmokeAlarm OverloadAlarm VideoStream
Check Smoke	Monitor the status of the smoke detector and generate an alarm if triggered	Passenger vehicles	SmokeAlarm	-
Check Overload	Monitor the status of the overload detector and generate an alarm if triggered	Passenger vehicles	OveloadAlarm	-

**Table 3.** Priority values associated to each task.

**Task**	**Node**	**Priority Level**	**Instances**	**Execution Type**
Get Status	All vehicles	Medium	1 (repeated in all vehicles)	Parallel
Get People Count	Passenger vehicles	Low	1 (repeated in all vehicles)	Parallel
Get Video Stream	Passenger vehicles	Lowest	N (one per passenger vehicle)	Individual
Process Alarms	Locomotive	High	1	Individual
Log Data	Locomotive	Low	1	Individual
Check Smoke	Passenger vehicles	High	N (one per passenger vehicle)	Parallel (not managed)
Check Overload	Passenger vehicles	High	N (one per passenger vehicle)	Parallel (not managed)

**Table 4.** Size and priority values associated to each data topic.

**Data Topic**	**Priority Level**	**Data Token Size (Bytes)**	Transmission Time (μs)*	Transmission Time (%EC)**
StatusData	High	3,000	240	2.4
PeopleCount	Medium	2	5.6	0.56
VideoStream	Low	250,000	20,000	200
SmokeAlarm	Highest	1	5.6	0.56
OverloadAlarm	Highest	1	5.6	0.56

* Time calculated for a 100 Mbps Ethernet LAN;

** Calculated with EC = 10 ms.
